# Inference of Genetic Diversity, Population Structure, and Selection Signatures in Xiangxi White Buffalo of China Through Whole-Genome Resequencing

**DOI:** 10.3390/genes15111450

**Published:** 2024-11-10

**Authors:** Chenqi Bian, Yang Luo, Jianbo Li, Huan Cheng, Fang He, Hongfeng Duan, Zulfiqar Ahmed, Chuzhao Lei, Kangle Yi

**Affiliations:** 1 Hunan Institute of Animal and Veterinary Science, Changsha 410131, China; 2 Key Laboratory of Animal Genetics, Breeding and Reproduction of Shaanxi Province, College of Animal Science and Technology, Northwest A and F University, Yangling 712100, China; 3 Department of Livestock and Poultry Production, Faculty of Veterinary and Animal Sciences, University of Poonch Rawalakot, Rawalakot 12350, Pakistan

**Keywords:** genetic diversity, population structure, selection signatures, whole-genome resequencing, Xiangxi white buffalo

## Abstract

(1) Background: Buffaloes are crucial livestock species for food and service in tropical and subtropical regions. Buffalo genetics, particularly in indigenous Chinese breeds such as the Xiangxi white buffalo (XWB), remains an intriguing area of study due to its unique traits and regional significance. (2) Methods: This investigation utilized the whole-genome sequences of twenty XWBs (newly sequenced), along with eighty published whole-genome sequences of other buffalo breeds (including Guizhou white buffalo, river buffalo, and Chinese buffalo in the Yangtze River). Using whole-genome sequencing analysis technology, the population structure, genomic diversity, and selection signatures of XWB were determined. (3) Results: This study revealed that the XWB, being phylogenetically positioned in the middle and lower reaches of the Yangtze River, exhibited substantial genomic diversity. Employing four selection sweep detection methods (CLR, iHS, π-ratio, and *F*_ST_), several genes were positively identified for adaptive traits in the XWB, including coat color phenotypes (*ASIP*, *KIT*), the nervous system (*GRIK2*), reproduction (*KCNIP4*), growth and development (*IFNAR1*, *BMP6*, *HDAC9*, *MGAT4C*, and *SLC30A9*), the body (*LINGO2*, *LYN*, and *FLI1*), immunity (*IRAK3* and *MZB1*), and lactation (*TP63*, *LPIN1*, *SAE1*). (4) Conclusions: In conclusion, this study enhances our understanding of the genetic distinctiveness and adaptive traits of XWB, highlighting selection signatures crucial for future breeding and conservation and ensuring sustainable use of this vital livestock resource.

## 1. Introduction

Domesticated buffaloes are found mainly in Asia. Based on differences in morphology, behavior, and chromosomal karyotype, domesticated buffaloes around the world are classified mainly into riverine (*Bubalus bubalis bubalis*, 2n = 50) and swamp buffaloes (*Bubalus bubalis carabanensis*, 2n = 48) [1]. Domesticated buffalo are important livestock in tropical and subtropical regions and play a pivotal role in providing meat and milk to communities. Buffalos produce more than 5% of the world’s total milk production [2] In addition, buffaloes are good draught animals that provide 20–30% of the labor force in terms of rice paddies [3].

White swamp buffaloes (a common variant of swamp buffaloes) have a white hair coat on their body that overlies pink skin [4]. The habitat of Guizhou white buffalo (GWB) is the northeastern part of Guizhou province, mainly concentrated in Fenggang and Wuchuan counties. Since the Qing Dynasty, the mentioned area has been the main breeding area for white buffaloes. And as a unique quality of the Hunan Xiangxi region, building upon the natural evolution of villages by environmental factors and local economic activities shapes the Xiangxi white buffalo (XWB) breed for numerous distinctive attributes such as a unique white coat, good conversion of poor roughages, resistance to tropical diseases, high bodily performance, and resilience in labor [4]. These traits have revealed XWB as a vital asset for the agricultural pursuits of residents in Xiangxi. However, comprehensive investigations of intricate genomic features and the selection of signature footprints for adaptive traits are lacking in the literature [2]. A whole-genome sequence (WGS)-based exploration may provide better understanding of unique genetic architecture and evolutionary dynamics in XWB.

With the development of WGS technology, the genetic structure, evolutionary history, origin, and domestication of livestock, such as costars, pigs, cattle, and sheep, have been sequenced [5]. It has been widely and systematically applied as an effective tool for determining genomic features in different livestock species [4].

Despite being a local specialty breed, XWB faces significant declines in population size, and overall breed quality, and difficulty in spreading genetic resources due to inadequate management and planning. While extensive research has been conducted on indigenous Chinese buffalo breeds such as the GWB, elucidating the genetic underpinnings of traits such as a white coat color [4], the XWB remains relatively unexplored.

In light of this research gap, this study used data from 20 XWBs, and 80 water buffalos discussed in a previous publication, to construct a set, aiming to comprehensively study genetic diversity, population structure, and selected genomic regions of XWB from a genome-wide perspective, and provide unique insights for XWB genetic resource conservation.

## 2. Materials and Methods

### 2.1. Ethics Statement

Approval was granted from the Experimental Animal Management Committee (EAMC) of Northwest A&F University (2011-31,101,684), in compliance with the National Standard of Laboratory Animals Guidelines for Ethical Review of Animal Welfare (GB/T 35892-2018) and the Guide for the Care and Use of Laboratory Animals: Eighth edition [6]. This study was approved by the Institutional Animal Care and Use Committee of Northwest A&F University (Permit number: NWAFAC1019).

### 2.2. Sample Collection and Genome Sequencing

A total of 20 XWB blood samples were collected from Xiangxi Tujia and Miao Autonomous Prefecture, Hunan Province, China (Supplementary Table S1). Genomic DNA was extracted by the standard phenol‒chloroform method [7], and NovaSeq sequencing was performed at the Institute of Novelty Bioinformatics, Beijing, China. In addition, we included 80 published whole-genome sequence data values from a public repository (SRA, https://www.ncbi.nlm.nih.gov/sra, accessed on 27 September 2022) database [8], including four buffalo populations, GWB (n = 30), river buffalo (RB) (n = 10), middle lower Yangtze (MLY) (Xinyang (n = 5), Poyanghu (n = 5), Fuzhong (n = 10)), and upper Yangtze (UY) (Guizhou (n = 7), Guangyuan (n = 5), Enshi (n = 4), and Yibin (n = 4)), which fully described the characteristics of population structure, genetic diversity, single-nucleotide polymorphisms (SNPs), and natural or artificial selection. The details of the samples used are presented in Supplementary Table S2.

### 2.3. Genome-Wide Alignment and Variation Detection

First, the clean reads were trimmed by using Trimmomatic (v0.38) [9]. After trimming, the remaining high-quality reads were aligned against the updated version of the buffalo reference genome (GWHAAJZ00000000) by using BWA-MEM (0.7.13-r1126) [10]. To obtain highly confident variants, we employed SAMtools (version 1.9) [10], Picard tools (http://broadinstitute.github.io/picard (accessed on 1 September 2023)), and the Genome Analysis Toolkit (GATK version 3.8-1-0-gf15c1c3ef) [11]. Based on the latest reference assembly (GWHAAJZ00000000), the SNPs were functionally annotated by using ANNOVAR (RRID:SCR_012821) [12].

### 2.4. Analysis of Genetic Structure and Relatedness of Buffalo Populations

The VCF files of 100 buffaloes were converted to PLINK format using VCFtools v0.1.12 (https://vcftools.github.io/index.html, accessed on 9 May 2023) software [13]. Chained loci were removed from the genomic data using PLINK software (version 1.9) through the parameter (--indep-pairwise 50 5 0.2) option [12]. For the genome-wide autosomal data, the genetic distance matrix between individuals was first calculated from the genotype information of SNP data using plink.distance.matrix, and the resulting file was subsequently opened in .meg format using MEGA7 and finally converted to the PLINK format using ITOL [14]. MEGA7 was used to open the resulting file in .meg format, after which the data were visualized using ITOL v5 (https://itol.embl.de/, accessed on 19 May 2023) [15]. Principal component analyses (PCAs) were performed using the EIGENSOFT 6.1.4 package in the SmartPCA [16] program for genome-wide autosomal SNP data, and scatter plots were drawn using the ggplot2 tool in RStudio software (version 4.3.2) to observe clustering relationships between the different populations. To explore ancestry ratios, we used ADMIXTURE software (version 1.3.0) to calculate genome-wide unlinked loci and estimate the ancestral composition of each individual [17]. This study was simulated from k = 2 to k = 4, and the results were visualized by RStudio software.

### 2.5. Genetic Diversity, Linkage Disequilibrium, and ROH Analysis

We estimated the nucleotide diversity of each species using VCFtools with a window size of 50 kb and a step size of 50 kb. The results were visualized using RStudio software [18]. PopLDdecay software (version 3.42) with default parameters was used to calculate r2 values for different populations to assess the degree of linkage disequilibrium (LD) in the populations, and the results were visualized using the R package (version 4.3.2) [19]. The length and number of contiguous pure fragments were calculated for each individual using PLINK software (version 1.9) [20]. The number and length of ROHs were estimated for each species, and the ROH lengths were classified into three categories: 0.5–1, 1–2, and 2–4 Mb, respectively [21].

### 2.6. Detection of Selection Signatures

We used the following methods to conduct a genome scan of XWB. First, we utilized nucleotide diversity (θπ) [22] and the composite likelihood ratio test (CLR) [23] to detect selective traits in the XWB. Nucleotide diversity was estimated by using VCFtools with a sliding window of 50 kb and a step size of 20 kb. CLRs for sites within a nonoverlapping 50 kb window were calculated using SweepFinder2 (version 1.0) [24].

Second, we compared XWB and RB using the fixation index (*F*_ST_) [22] and cross-population extended haplotype purity (XP-EHH) [25]. The *F*_ST_ analysis was performed using VCFtools in a 50 kb window with 20 kb steps [26]. XP-EHH statistics were based on extended haplotypes and calculated for each population using Selscan v1.1 [27]. For the XP-EHH selectivity scan, our test statistic was the mean normalized XP-EHH score for each 50 kb region. XP-EHH scores were directional: positive scores indicated that selection may have occurred in XWB, while negative scores indicated the same for the reference population. Significant genomic regions were identified by *p* values < 0.01. Genomic regions identified by at least two methods were considered candidates for positive selection [18].

### 2.7. Enrichment Analyses of Candidate Genes

To better understand the gene functions and signaling pathways of the identified candidate genes, we performed gene ontology (GO) and Kyoto Encyclopedia of Genes and Genomes (KEGG) pathway enrichment analyses using KOBAS 3.0 [28]. GO and KEGG pathway enrichment was considered to be significant when the corrected *p* value was < 0.05.

## 3. Results

### 3.1. Whole-Genome Sequencing and SNP Identification

The twenty XWBs with clean data ranging from 203,987,400 to 241,759,504 reads, with an average sequencing depth of ~12.51X (Supplementary Table S1), were genotyped with the open genomes of 70 Chinese swamp buffaloes and 10 river buffaloes via BWAMEM suite software (version 0.7.13-r1126). While comparing the obtained results with the buffalo reference genome (GWHAAJZ00000000), a 98.33% alignment ratio and an average sequencing depth of 10.11X were observed. The chromosome change rate of the buffalo collection was also measured (Supplementary Table S3). We annotated 93,282,576 biallelic SNPs found in 100 buffaloes (Supplementary Table S4). Genome annotation revealed that most of the SNPs in the XWB were located in intronic (38.59%) or intergenic (58.14%) regions.

### 3.2. Population Genetic Structure and Ancestral Relationships

**Figure 1:**
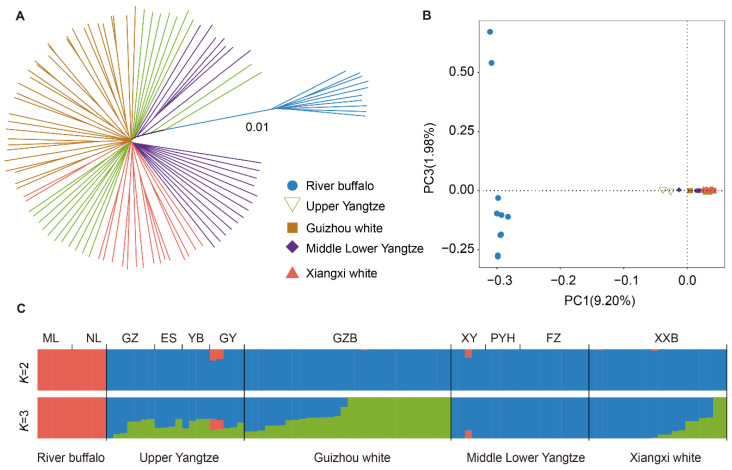
Population genetic analysis of buffalo. (**A**) Neighbor-joining tree of buffaloes. (**B**) Principal component analysis of buffaloes with PC1 (9.20%) and PC3 (1.98%). (**C**) Genetic structure of buffaloes using ADMIXTURE with K = 2 and K = 3.

The neighbor-joining (NJ) phylogenetic tree was constructed based on the whole-genome data of 100 buffaloes as shown in Figure 1A; different colored branches and clades represent buffaloes from different regions. The buffaloes were divided into two main branches: swamp and riverine buffaloes. XWBs are positioned as swamp buffaloes and share close genetic identity with the MLY and UY buffaloes. PCA revealed that PC1 separated swamp from riverine buffaloes, and PC3 revealed a difference within swamp buffaloes (Figure 1B). The results of the PCA confirmed the NJ-tree phylogenetic patterns. The ancestral component analysis of the whole-genome data of 100 buffaloes using ADMIXTURE software (version 1.3.0) showed a minimum CV when K = 2, meaningfully indicating that the ancestral fragment components of the XWB and GZB populations were the same. When K = 3, two kinds of ancestral component ratios were shown within the swamp-type buffalo population, and an obvious difference was observed between UY and MLY buffalo groups. In the Yangtze River basin, the swamp-type buffaloes’ lineage purity increases as we move closer to the lower reaches. This pattern aligns with the phylogenetic tree and principal component analysis (PCA), supporting the existing conclusion about genetic exchange between the two buffalo species (Figure 1C).

### 3.3. Patterns of Genomic Variation

**Figure 2:**
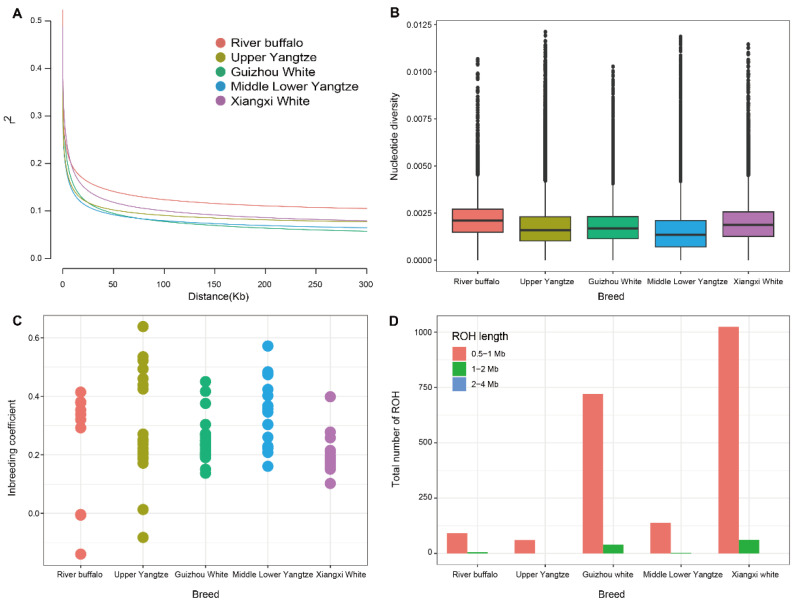
Population genetic diversity of buffalo. (**A**) Average genome-wide LD attenuation in different varieties. (**B**) Box plots of nucleotide diversity for each variety. (**C**) Inbreeding coefficient of each individual. (**D**) ROH distribution across chromosomes for each breed.

By calculating the LD between paired SNPs in the range of 1–300 kb intervals for each breed, as shown in Figure 2A, the genome-wide average LD decay was found to be lowest in GWB, and high in XWB. Different breeds within the same population type had different decay rates of LD due to their different genetic backgrounds. Domestication selection reduces the genetic diversity of populations and strengthens the correlation between loci. Thus, in general, the greater the degree of domestication and the greater the intensity of selection, the slower the rate of LD decay [29]. In terms of genomic nucleotide diversity, XWB had greater nucleotide diversity, second only to RB (Figure 2B). The average inbreeding coefficient of the XWB population was low (Figure 2C). To assess the run of homozygosity (ROH) patterns of XWB and other buffalo breeds, we categorized the length of the ROH into 0.5–1, 1–2, and 2–4 Mb. The shorter ROH fragments of XWB were the most common, and the white buffalo population had more and longer ROH fragments than did the other buffaloes (Figure 2D).

### 3.4. Patterns of Selection

**Figure 3:**
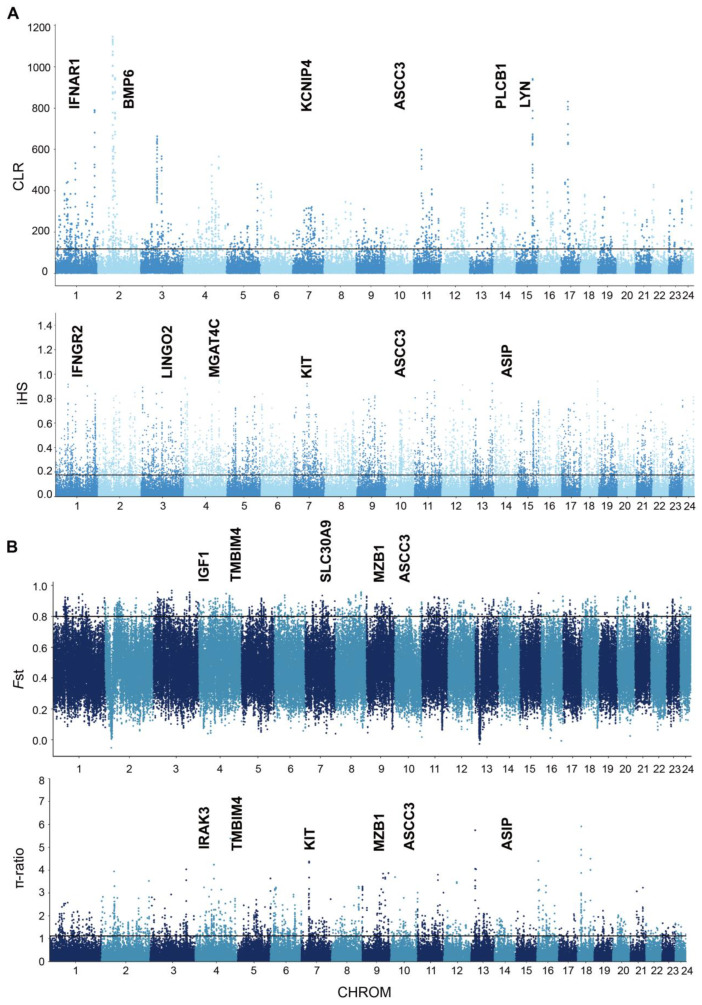
Analysis of genome positive selection characteristics of Xiangxi white buffalo. (**A**) Manhattan plot of selective sweeps by CLR and iHS methods in XWB. (**B**) Manhattan plot of selective sweeps by *F*_st_ and π-ratio methods between XWB and RB.

**Figure 4:**
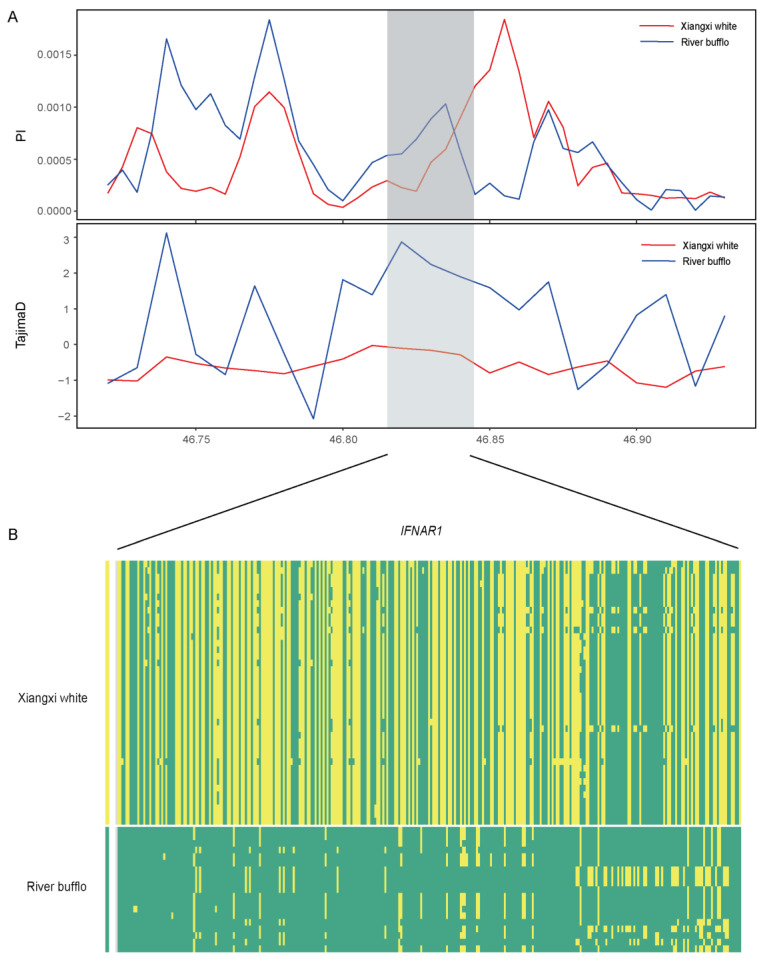
Candidate genes involved in positive selection. (**A**) Tajima’s *D* plot of *IFNAR1* gene. (**B**) Haplotype diversity of *IFNAR1* gene.

We used CLR and iHS to test the selection-related genomic regions of the XWB population (Figure 3A). According to the statistics of the CLR (Supplementary Table S5, Supplementary Figure S1A), we obtained 820 putative genes, while 2790 putative genes were obtained by the iHS test (Supplementary Table S6, Supplementary Figure S1A); these included genes related to reproduction (*BMP6*, *ADCY3*, *KCNIP4*, *IFNAR1*, and *IFNAR2*), meat quality (*MEF2C* and *PLCB1*), and heat resistance genes (*ADAMTSL1* and *PLCB1*). Notably, *IFNAR1* was screened by both methods, and further, based on the haplotype of the *IFNAR1* gene, its performance in the two populations significantly differed (Figure 4A,B). *IFNAR1* can bind to interferon-tau (IFNT), which is an anti-luteolytic factor secreted by the trophoblast during pregnancy, to signal or stimulate the expression of certain factors to establish maternal recognition and maintain pregnancy [30]. The overlapping genes were analyzed for enrichment (Supplementary Table S7). The KEGG pathway enrichment analysis revealed that cholinergic synapses (*p* < 0.05) and morphine addiction (*p* < 0.05) were associated with nerve conduction. The GO enrichment analysis (Supplementary Figure S2A) revealed that several GO terms were related to motor nerves and muscles, including the “glutamatergic synapse” (corrected *p* value = 0.006), “nervous system process” (corrected *p* value = 0.035), “GABA-ergic synapse” (corrected *p* value = 0.035), “neurotransmitter secretion” (GO:0007269, corrected *p* value = 0.011), etc.

To better understand the differential selection signals between XWB and RB, *F*_ST_ and π-ratios were calculated (Figure 3B), and 535 (Supplementary Table S8, Supplementary Figure S1B) and 685 genes (Supplementary Table S9, Supplementary Figure S1B) related to hypothetical favorable positive selection were obtained, including coat color (*ASIP* and *KIT*) and meat quality (*PTK2* and *ROCK2*), respectively. The overlapping regions detected by both methods were considered candidate regions, and a total of 85 candidate genes were found to overlap for a subsequent enrichment analysis (Supplementary Table S10). Notably, the GO pathway enrichment results (Supplementary Figure S2B) revealed that the “negative regulation of transcription by RNA polymerase II” (corrected *p* value = 1.23 × 10^−5^), “RNA polymerase II cis-regulatory region sequence-specific DNA binding” (corrected *p* value = 0.0007), and “positive regulation of transcription by RNA polymerase II” (corrected *p* value = 0.0009) are related to RNA polymerase II, which contains *SOX6*, *SOX30*, *STAT6*, *RORC*, and *GLI3* genes, associated with muscle growth. The *IFNAR1* gene, which was identified as being associated with a response to interferon-alpha biological processes, is reported to be expressed in bovine placental tissue during early pregnancy and is associated with immune tolerance protection in the mother and fetus [30].

Through the above four methods, a total of eight overlapping genes were obtained; *CSMD1* was related to mammary gland development and the expression of progesterone receptors, *PDE1A* was involved in muscle development, *GART* associated with strong pathogenicity, and the *ITSN1* applied to important embryonic development. The *CSMD1* gene plays an important role in mammary gland development. Studies have shown that the *CSMD1* gene can increase the expression of progesterone receptors (PRs) [31], which are a major regulator of female reproductive tissue and can control development, proliferation, and differentiation during the reproductive cycle and pregnancy. In addition, the *PDE1A* gene has been confirmed to be associated with muscle development in cattle, a mutation in the *GART* gene is associated with strong pathogenicity in cattle, and the *ITSN1* gene is considered to be an important embryonic gene.

## 4. Discussion

Population genetic diversity is important for the conservation of species evolution. A comprehensive understanding of population structure and genetic diversity is essential for facilitating the environmental adaptation, optimal utilization, and effective protection of genetic resources within buffalo breeds [18,32]. This imperative serves as a foundational principle in ensuring the sustainable management and preservation of these valuable genetic assets [33]. Nucleotide diversity is the most important factor affecting genetic diversity [34]. A single environment and high-intensity artificial breeding may reduce nucleotide diversity [34]. The nucleotide diversity (mean π = 0.00197) of the XWB buffaloes was greater than that of other buffaloes in China, which may be related to the weak selection history. For an individual, a region in its genome in which all loci are pure is called a run of homozygosity (ROH). We used the ROH to assess the purity of each sample [20]. The distribution of the ROH of XWB was analyzed via comparison with that of other buffalo species. ROH is very common in buffalo autosomes [35]. Due to differences in population structure, effective population size, mating system, and selection intensity, the number, length, distribution, and frequency of ROH in the population genome may vary [36]. Compared with other buffalo breeds in this study, XWB had the most short/medium (0.5–2 Mb) ROH.

Investigating the correlation between population structure and phylogeny holds significant importance in comprehending historical statistical patterns of populations and tracing ancestral information. In this study, the complete genome sequences of 20 XWBs were analyzed. Through a comprehensive analysis, it has been demonstrated that the XWB belongs to the Yangtze River basin, aligning with its geographical location. Furthermore, the genetic makeup of water buffaloes in China’s Yangtze River basin closely correlates with their geographic origin.

Wild buffaloes are known to be very fierce animals, but domesticated swamp buffaloes are much tamer [37]. The KEGG pathway enrichment analysis of genes in the selective clearance region of XWB revealed many pathways related to nerve conduction and behavior, among which the most significant pathway was the synaptic vesicle cycle. According to previous reports, gamma-aminobutyric acid (GABA) is an important inhibitory neurotransmitter in the central nervous system, and when GABA is lacking or when GABA neurons are abnormal, anxiety, fatigue, and other states develop [38]. The synthesis and packaging of GABA are important steps in the synaptic vesicle circulation pathway. GABA is produced by glutamate decarboxylase and subsequently transported to synaptic vesicles. When released, GABA within synaptic vesicles is kept at a high concentration for use. In addition, the GO pathway analysis revealed significant enrichment of many genes related to nervous system development, such as genes related to neurons, dendrites, and synapses. This enrichment may be related to the development of docile characteristics in swamp buffalo. The *SLC6A1* gene encodes a transporter of GABA localized to the plasma membrane [39]. The *ADCY8* gene catalyzes cAMP formation in response to calcium entry, leading to cAMP signaling activation, which affects processes such as synaptic plasticity and insulin secretion [40]. *SLC5A7* encodes presynaptic sodia-dependent high-affinity choline transporter 1, which takes up choline to presynaptic nerve endings after acetylcholine is broken down by acetylcholinesterase in the synaptic space [41].

By comparison with those in RB, we found that XWB has a unique signaling pathway related to the immune system and muscle growth. In this study, the KEGG and GO pathways associated with muscle development were significantly enriched, and the most significantly enriched pathway associated with muscle development was ATP binding, which included the genes *MYH10*, *MYH9*, *MYLK3*, *PRKAA1*, and *ROCK2*. Previous studies have confirmed that the *ROCK2* gene is associated with meat quality traits in cattle. This finding is consistent with the findings in the mountainous area of XWB that had better draft and meat performance, which may be the result of long-term artificial selection [42].

There were genetic differences between river-type and swamp-type buffalo, which were reflected in the haplotype heat map of the selected *IFNAR1* gene (Figure 4). The IFNAR1 gene is located on chromosome 1 of water buffalo and its length is about 0.029 M bp. Tajima’s *D* analysis showed that the value of XWB decreased significantly, indicating a strong positive selection of the *IFNAR1* gene in XWB. In addition, a white fur color is a common phenotypic trait in mammals. Current studies on white buffalo hair color have confirmed that a 2809 bp long linear 1 insertion in the *ASIP* (Agouti signaling protein) gene is a disease-causing mutation in the swamp buffalo white hair phenotype [4]. According to the four selection methods used in this study, *ASIP*, *KIT*, and other genes were confirmed to be related to cattle hair color through iHS and π-ratio analyses, which is the reason for the white color of the XWB.

## 5. Conclusions

In summary, our whole-genome scan of XWB revealed its unique genetic makeup, highlighting its strong diversity and adaptive qualities. The identified genes linked to the coat color, nervous system, reproduction, growth, body, immunity, and lactation underscore the importance of the breed. These genetic markers are guideposts for future research, enriching our grasp of buffalo genetics and aiding informed choices for sustainable agriculture and biodiversity conservation.

## Data Availability

Sequences are available from NCBI with the Bioproject accession number PRJNA1166923.

## Supplementary Materials

The following supporting information can be downloaded at: https://www.mdpi.com/article/10.3390/genes15111450/s1, Table S1: List of buffalo samples for analysis of genetic information. Table S2: Summary of additional buffalo sample information. Table S3: Change rate by chromosome. Table S4: Functional annotation of identified single-nucleotide polymorphisms (SNPs) using ANNOVAR. Table S5: A summary of genes from CLR in Xiangxi white buffalo. Table S6: A summary of genes from iHS in Xiangxi white buffalo. Table S7: KEGG PATHWAY and Gene Ontology of Xiangxi white buffalo candidate genes overlapped by CLR and iHS methods. Table S8: A summary of genes from Fst in Xiangxi white buffalo (compared to river buffalo). Table S9: A summary of genes from θπ in Xiangxi white buffalo (compared to river buffalo). Table S10: KEGG PATHWAY and Gene Ontology of Xiangxi white buffalo candidate genes overlapped by *F*_st_ and θπ methods. Figure S1: The number of candidate genes supported by the selection methods in each Venn diagram component.(A) The overlap results of genes screened by CLR and iHS in XWB. (B) The overlap results of genes screened by FST and π-ratios between XWB and RB.; Figure S2: Results of GO enrichment analysis of overlapping genes. (A) The top ten pathways after GO enrichment were obtained by overlapping genes obtained by CLR and iHS. (B) The top ten pathways after GO enrichment were obtained by overlapping genes obtained by FST and π-ratios.

## References

[1] Fischer H., Ulbrich F. (1967). Chromosomes of the Murrah buffalo and its crossbreds with the asiatic swamp buffalo (Bubalus bubalis). J. Animal Breed. Genet..

[2] Windsor P., Martin S., Khounsy S., Young J., Thomson P., Bush R. (2021). Improved milk production from supplementation of Swamp Buffalo with molasses nutrient blocks containing 10% urea. Dairy.

[3] Michelizzi V.N., Dodson M.V., Pan Z., Amaral M.E.J., Michal J.J., McLean D.J., Womack J.E., Jiang Z. (2010). Water buffalo genome science comes of age. Int. J. Biol. Sci..

[4] Liang D., Zhao P., Si J., Fang L., Pairo-Castineira E., Hu X., Xu Q., Hou Y., Gong Y., Liang Z. (2021). Genomic Analysis Revealed a Convergent Evolution of LINE-1 in Coat Color: A Case Study in Water Buffaloes (*Bubalus bubalis*). Mol. Biol. Evol..

[5] Kalds P., Zhou S., Chen Y., Wang X. (2024). Modeling animal genomics in mice: An authentic approach for the functional interrogation of evolutionarily and agriculturally critical variants. Anim. Res. One Health.

[6] (2018). Laboratory Animal—Guideline for Ethical Review of Animal Welfare.

[7] Green M.R., Sambrook J. (2012). “Molecular Cloning: A Laboratory Manual”, in the Three-Volume Set.

[8] Leinonen R., Sugawara H., Shumway M., on behalf of the International Nucleotide Sequence Database Collaboration (2010). The sequence read archive. Nucleic Acids Res..

[9] Bolger A.M., Lohse M., Usadel B. (2014). Trimmomatic: A flexible trimmer for Illumina sequence data. Bioinformatics.

[10] Li H., Durbin R. (2009). Fast and accurate short read alignment with Burrows‒Wheeler transform. Bioinformatics.

[11] Li H., Handsaker B., Wysoker A., Fennell T., Ruan J., Homer N., Marth G., Abecasis G., Durbin R. (2009). The Sequence Alignment/Map format and SAMtools. Bioinformatics.

[12] Wang K., Li M., Hakonarson H. (2010). ANNOVAR: Functional annotation of genetic variants from high-throughput sequencing data. Nucleic Acids Res..

[13] Purcell S., Neale B., Todd-Brown K., Thomas L., Ferreira M.A.R., Bender D., Maller J., Sklar P., de Bakker P.I.W., Daly M.J. (2007). Plink: A tool set for whole-genome association and population-based linkage analyses. Am. J. Hum. Genet..

[14] Kumar S., Stecher G., Tamura K. (2016). MEGA7: Molecular Evolutionary Genetics Analysis Version 7.0 for Bigger Datasets. Mol. Biol. Evol..

[15] Letunic I., Bork P. (2021). Interactive Tree Of Life (iTOL) v5: An online tool for phylogenetic tree display and annotation. Nucleic Acids Res..

[16] Patterson N., Price A.L., Reich D. (2013). Population structure and eigenanalysis. PLoS Genet..

[17] Alexander D.H., Novembre J., Lange K. (2009). Fast model-based estimation of ancestry in unrelated individuals. Genome Res..

[18] Chen Z., Zhu M., Wu Q., Lu H., Lei C., Ahmed Z., Sun J. (2023). Analysis of genetic diversity and selection characteristics using the whole-genome sequencing data of five buffaloes, including Xilin buffalo, in Guangxi, China. Front. Genet..

[19] Zhang C., Dong S.S., Xu J.Y., He W.M., Yang T.L. (2019). PopLDdecay: A fast and effective tool for linkage disequilibrium decay analysis based on variant call format files. Bioinformatics.

[20] Makanjuola B.O., Maltecca C., Miglior F., Marras G., Abdalla E.A., Schenkel F.S., Baes C.F. (2021). Identification of unique ROH regions with unfavorable effects on production and fertility traits in Canadian Holsteins. Genet. Sel. Evol..

[21] Forutan M., Ansari M.S., Baes C., Melzer N., Schenkel F.S., Sargolzaei M. (2018). Inbreeding and runs of homozygosity before and after genomic selection inNorth American Holstein cattle. BMC Genom..

[22] Hudson R. (1992). Estimation of levels of gene flow from DNA sequence data. Genetics.

[23] Nielsen R., Williamson S., Kim Y., Hubisz M.J., Clark A.G., Bustamante C. (2005). Genomic scans for selective sweeps using SNP data. Genome Res..

[24] DeGiorgio M., Huber C.D., Hubisz M.J., Hellmann I., Nielsen R. (2016). SweepFinder2: Increased sensitivity, robustness and flexibility. Bioinformatics.

[25] Sabeti P.C., Varilly P., Fry B., Lohmueller J., Hostetter E., Cotsapas C., Xie X., Byrne E.H., McCarroll S.A., Gaudet R. (2007). Genome-wide detection and characterization of positive selection in human populations. Nature.

[26] Danecek P., Auton A., Abecasis G., Albers C.A., Banks E., DePristo M.A., Handsaker R.E., Lunter G., Marth G.T., Sherry S.T. (2011). The variant call format and VCFtools. Bioinformatics.

[27] Szpiech Z.A., Hernandez R.D. (2014). Selscan: An efficient multithreaded program to perform EHH-based scans for positive selection. Mol. Biol. Evol..

[28] Shen S., Kong J., Qiu Y., Yang X., Wang W., Yan L. (2019). Identification of coregenes and outcomes in hepatocellular carcinoma by bioinformatics analysis. J. Cell Biochem..

[29] El H.A., Rocha D., Venot E., Blanquet V., Philippe R. (2021). Long-range linkage disequilibrium in French beef cattle breeds. Genet. Sel. Evol..

[30] Wang W., Liu R., Liang X., Zhao Q., Qu P., Yao K., Jiang M., Luo Y., Zhang W., Qing S. (2018). Expression of IFNAR1 and IFNAR2 in cattle placenta during early pregnancy. Reprod. Domest. Anim..

[31] Doyle J.L., Berry D.P., Veerkamp R.F., Carthy T.R., Evans R.D., Walsh S.W., Purfield D.C. (2020). Genomic regions associated with muscularity in beef cattle differ in five contrasting cattle breeds. Genet. Sel. Evol..

[32] Chen Y., Wang P., He X., Liu Y., Chu M. (2024). SEMA4G targeted by miR-363-5p regulates the proliferation of granulosa cells in Yunshang black goats. Anim. Res. One Health.

[33] Luo X., Li S., Liu Y., Ahmed Z., Wang F., Liu J., Zhang J., Chen N., Lei C., Huang B. (2022). Assessing the Role of Ancestral Fragments and Selection Signatures by Whole-Genome Scanning in Dehong Humped Cattle at the China–Myanmar Border. Biology.

[34] Li S., Lei H., Li J., Sun A., Ahmed Z., Duan H., Chen L., Zhang B., Lei C., Yi K. (2023). Analysis of genetic diversity and selection signals in Chaling cattle of southern China using whole-genome scan. Anim. Genet..

[35] Macciotta N.P.P., Colli L., Cesarani A., Ajmone-Marsan P., Low W.Y., Tearle R., Williams J.L. (2021). The distribution of runs of homozygosity in the genome of river and swamp buffaloes reveals a history of adaptation, migration and crossbred events. Genet. Sel. Evol..

[36] Ceballos F.C., Joshi P.K., Clark D.W., Ramsay M., Wilson J.F. (2018). Runs of homozygosity:windows into population history and trait architecture. Nat. Rev. Genet..

[37] Sun T., Shen J., Achilli A., Chen N., Chen Q., Dang R., Zheng Z., Zhang H., Zhang X., Wang S. (2020). Genomic analyses reveal distinct genetic architectures and selective pressures in buffaloes. GigaScience.

[38] Nuss P. (2015). Anxiety disorders and GABA neurotransmission: A disturbance of modulation. Neuropsychiatr. Dis. Treat..

[39] Palmer S., Towne M.C., Pearl P.L., Pelletier R.C., Genetti C.A., Shi J., Beggs A.H., Agrawal P.B., Brownstein C.A. (2016). SLC6A1 Mutation and Ketogenic Diet in Epilepsy With Myoclonic-Atonic Seizures. Pediatr. Neurol..

[40] Zhang M., Wang H. (2023). Ca^2+^-stimulated ADCY1 and ADCY8 regulate distinct aspects of synaptic and cognitive flexibility. Front. Cell Neurosci..

[41] Cruz P.M.R., Hughes I., Manzur A., Munot P., Ramdas S., Wright R., Breen C., Pitt M., Pagnamenta A.T., Taylor J.C. (2021). Presynaptic congenital myasthenic syndrome due to three novel mutations in SLC5A7 encoding the sodium-dependant high-affinity choline transporter. Neuromuscul. Disord..

[42] Chen Y., Guo Y., Ge F., Gao H., Zhou J., Wu X., Qian C., Wang Z., Wang Z., Zhu B. (2024). Developing a liquid capture chip to accelerate the genetic progress of cattle. Anim. Res. One Health.

