# Supplementary material for: Inference of Genetic Diversity, Population Structure, and Selection Signatures in Xiangxi White Buffalo of China Through Whole-Genome Resequencing
Source: Genes (Basel). 2024 Nov 10;15(11):1450. doi: 10.3390/genes15111450 (PMC11594040; doi:10.3390/genes15111450)
Supplement: Supplementary file 1 [file genes-15-01450-s001.zip › Supplementary Figure.docx]

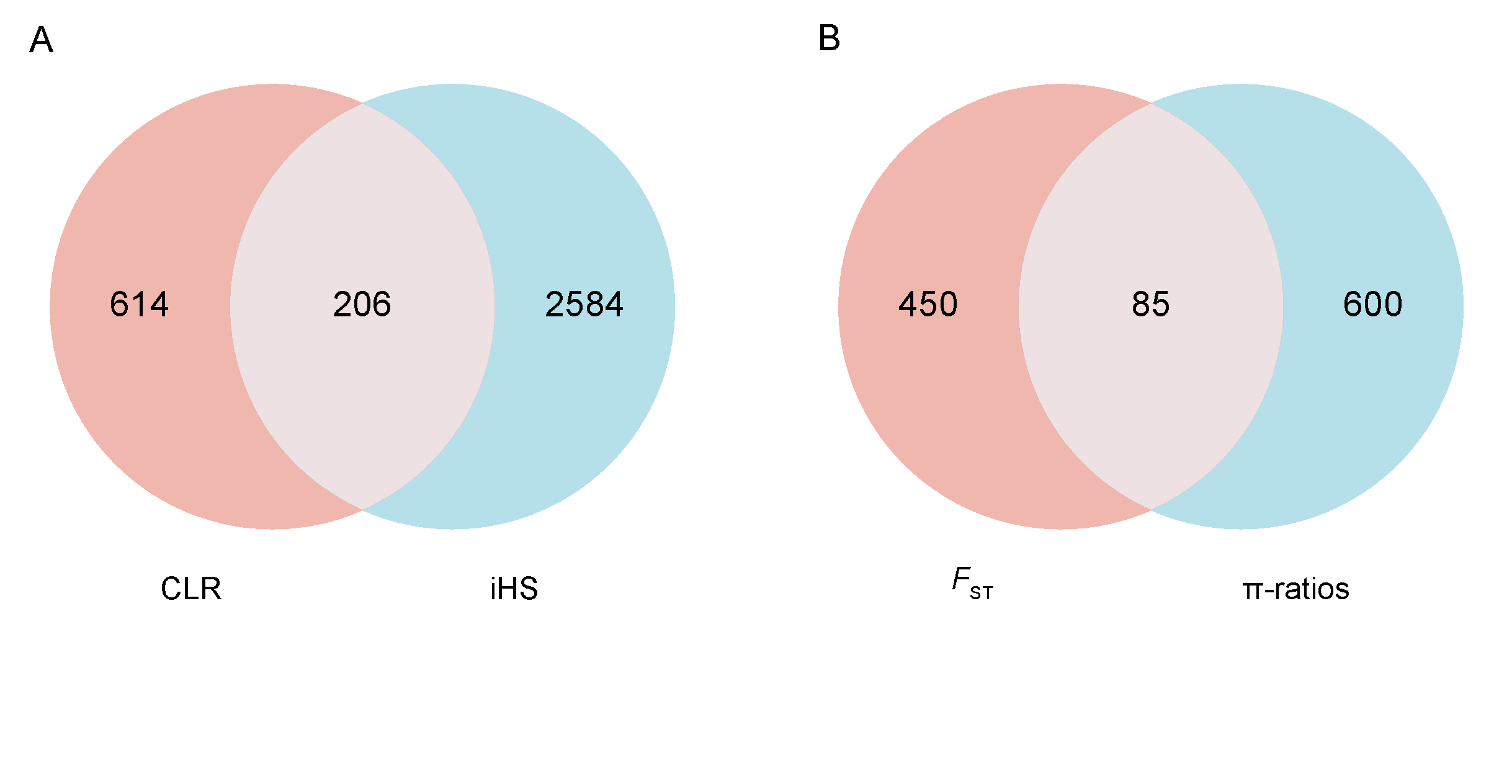


Supplementary Figure S1. The number of candidate genes supported by the selection methods in each Venn diagram component.(A) The overlap results of genes screened by CLR and iHS in XWB. (B) The overlap results of genes screened by *F*_ST_ and π-ratios between XWB and RB.


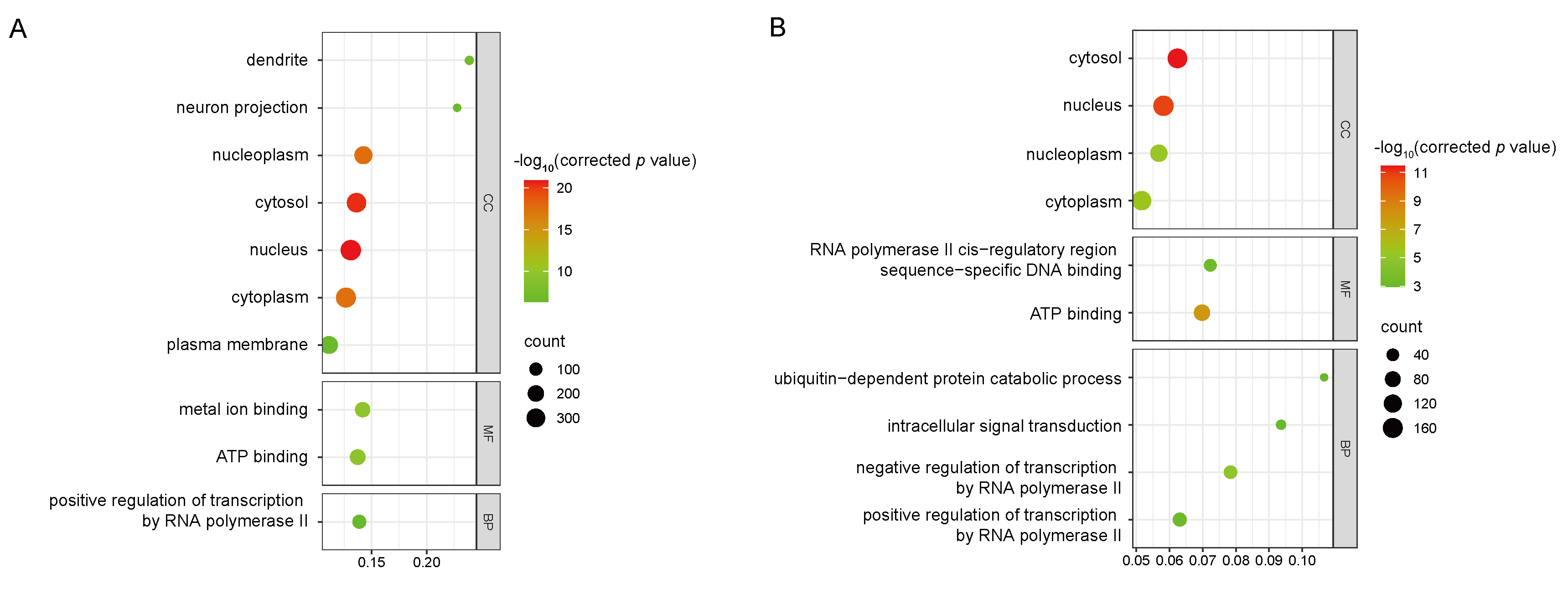


Supplementary Figure S2. Results of GO enrichment analysis of overlapping genes. (A) The top ten pathways after GO enrichment were obtained by overlapping genes obtained by CLR and iHS. (B) The top ten pathways after GO enrichment were obtained by overlapping genes obtained by *F*_ST_ and π-ratios.
